# A review of Japanese-style bathing: its demerits and merits

**DOI:** 10.1186/s40101-022-00278-0

**Published:** 2022-02-15

**Authors:** Yutaka Tochihara

**Affiliations:** Department of Human Science, Faculty of Design, Kyushu University, 4-9-1 Shiobaru, Minami-ku, Fukuoka, 815-8540 Japan

**Keywords:** Japanese-style bathing, Sudden death, Elderly, Blood pressure, Cold, Sleep

## Abstract

Japanese-style bathing (JSB), which involves soaking in hot water up to the shoulders in deep bathtubs for a long time in the evening to night, is unique. Many experimental and epidemiological studies and surveys have shown that JSB improve sleep quality, especially shortens sleep onset latency in winter. In addition, repeated JSB lead the improvement of depressive symptoms. JSB is a simple and low-cost non-pharmacological measure to sleep difficulty in winter and mental disorders, especially for the elderly. On the contrary, drowning, while soaking in a bathtub, is the most common of accidental death at home in Japan. It is estimated that approximately 19,000 Japanese individuals die annually while taking a bath, mostly during winter, and most victims are elderly people. Elderly Japanese people tend to prefer a higher-risk JSB because the temperature inside the house during winter, especially the dressing room/bathroom temperature, is very low. Since the physiological thermal effect of the elderly associated with bathing is relatively lower among the elderly than the young, the elderly prefer to take a long hot bath. This elderly’s favorite style of JSB results in larger increased blood pressure in dressing rooms and larger decreased in blood pressure during hot bathing. A sudden drop in blood pressure while immersed in the bathtub leads to fainting and drowning. Furthermore, elderly people are less sensitive to cold air or hot water, therefore, it is difficult to take appropriate measures to prevent large fluctuations in blood pressure. To ensure a safe and comfortable winter bathing, the dressing room/bathroom temperature needs to be maintained at 20 °C or higher, and several degrees higher would be recommended for the elderly.

## Background

### Japanese-style bathing

Bathing styles vary worldwide, including bathtub bathing, sauna bathing, and showering. Each country has its own bathing style, such as the Finnish sauna [1, 2] and Turkish bath [3]. Japanese-style bathing (JSB), which involves soaking in hot water up to the shoulders in deep bathtubs for a long time in the evening to night, is unique and different from Western-style bathing. In the West, the water used for bathing is less hot, and people soak their chest in a long bathtub. In the west, people tend to wash their body in the bathtub, while Japanese people tend to have a washing space outside the bathtub [4–6]. One of the reasons why they clean their body outside the tub is that Japanese do not normally change the water between each bath.

Japanese people like bathing very much. Tokyo Gas Inc. investigated the bathing style of 2600 people (15–75 years) in the Tokyo metropolitan area, and reported that 85.7% of people liked soaking in a hot water bathtub [7]. Approximately 70% of people take a bath daily in the winter. The reasons why they like bathing are as follows: warming the body (71.2%), recover from fatigue (67.2%), relax the body (65.9%), refresh their feelings (37.2%), and be able to sleep well (35.8%).

### History of Japanese bathing

In Japan, until the middle of the Edo period (1600–1864), bathing by storing steam in a room (steam bath) was common and had religious implications. Bathing through immersion of the body in hot water (hot water public bath) became widespread during the late eighteenth century. Home baths was widely practiced in the middle of the twentieth century [6, 8]. The cultural anthropologist, Ruth Benedict, in her book entitled *“The Chrysanthemum and the Sword”* published in 1946, provided an explanation of sleep, food, love, and sake in the chapter “The circle of human feelings,” and described the characteristics of the Japanese hot bath in detail [9]. “*One of the best loved minor pleasures of the body in Japan is the hot bath. For the poorest rice farmer and the meanest servant, just as much as for the rich aristocrat, the daily soak in superlatively heated water is a part of the routine of every late afternoon. The commonest tub is a wooden barrel with a charcoal fire under it to keep the water heated to 110° Fahrenheit and over. People wash and rinse themselves all over to before they get into the tub and then give themselves over to their enjoyment of warmth and relaxation of soaking. They sit in the bath with their knees drawn up in fetal position, the water up to their chins*” [9].

There are various theories regarding why Japanese people prefer hot water baths in winter and take baths almost every day. First, indoor in Japanese houses are generally cold in winter [10, 11]. In winter, to prevent feeling of cold after bathing, it may be necessary to soak in a hot water to stay warm [5]. Conventional Japanese houses have a structure that surpasses the heat and humidity of the summer. The ease of spending summer has long been prioritized in Japan. In the famous book *“Essays in Idleness*” by Kenko Yoshida in the Kamakura period (890–910), “*A house should be built with the summer in mind. In winter it is possible to live anywhere, but a badly made house is unbearable when it gets hot*.” [12]. Second, Japan has numerous water resources; hence, water supply is relatively cheap [13], so Japanese people do not hesitate in pouring hot water in the bathtub. Finally, there are many hot springs in Japan, increasing the opportunities to take hot spring baths, which may also be related factor.

### Sudden death during bathing

By contrast, JSB in winter can causes serious health problems as well. It has been reported that there are many sudden deaths during JSB [5, 14–23]. Takahashi et al. [22] and Suzuki et al. [15] estimated that the annual number of deaths during bathing in Japan was 17,000 and 19,000, respectively. Most cases occurred in winter, and the victims were mostly elderly people aged 65 and over. In Japan, the elderly population ratio account for 29.1% in 2021, and this ratio is expected to increase to 33.4% in 2035. Therefore, if effective measures are not taken, it has been warned that bathing deaths will reach 27,000 annually [15]. In Japan, which has reached a super-aged society, it is important to reduce the number of accidents related to bathing among the elderly population from the viewpoint of physiological anthropology.

This review aimed to summarize the effects of JSB on the human body. The effects were divided into two categories: demerits and merits. There are many benefits to JSB, such as warming of the body, recovery from fatigue, a refresh feeling, and enhanced sleep. In this review, we focused on evaluating the efficiency of JSB in improving sleep and depressive symptoms in winter. The demerit of JSB is sudden death during bathing of the elderly in winter. This review clarified why elderly people become victims during bathing in winter by conducting various physiological and psychological experiments and surveys on the effects of JSB, and also mention countermeasures against bathing accidents.

## Demerits of JSB

### Accidental deaths at home

Table 1 shows the number of accidental deaths at home announced by the Japanese Ministry of Health, Labor and Welfare 2019 [24]. A total number was 13,800 Japanese individuals die due to household accidents, of whom 11,987 (86.9%) were 65 years old or older. “Drowning” (41.1% of the total) was the most common cause of accidental death at home in the same year, followed by “asphyxiation” and “falls.” “Drowning” was a summer accident in the sea bathing and pool long ago, but recently Matsui and Kagamimori [17] reported that many deaths occurred “in the bathtub (60%),” of which 89% occurred at home, by age group, the number of elderly people who die during bathing is particularly high (85%). Moreover, Suzuki et al. [23] investigated bath-related deaths from all cases handled by the Tokyo Medical Examiner’s Office from 2009 to 2011. The autopsy findings revealed that water inhalation was the primary cause of death in majority of the cases (79.1%).

**Table 1:** Number of accidental deaths at home in Japan in 2019

	Total	Over 65 years	(%)
Deaths in houses	13,800	11,987	86.9
Asphyxiation	3187	2747	86.2
**Death by drowning**	**5673**	**5310**	**93.6**
Death by fire	813	602	74.0
Death by falls	2394	2088	87.2
Others	1733	1240	71.6
Deaths by traffic accident	4279	2508	58.6

However, it is often reported that the cause of death during bathing is not only “drowning” but may also be heart disease, cerebrovascular disease, etc. The number of drowning deaths shown in the vital statistics of the Ministry of Health, Labor, and Welfare does necessarily indicate the number of deaths associated with bathing. Concerns were raised as the statistical provided did not represent the entire number of deaths from bathing. Therefore, the cases in which ambulances were requested to the fire departments in several prefectures were considered to clarify the reality of related fatal accidents [19]. As a result, “cardiopulmonary arrest,” “heart disease,” or “cerebrovascular disease,” was often indicted as the cause of death in the death certificates of victims who died while taking a bath, “drowning” is rarely indicated as the cause of death in this patient group.

Takahashi et al. [22] of the Tokyo Metropolitan Institute of Health and Longevity Medical Center surveyed 634 fire departments nationwide, and determined that a total of 9360 elderly people experienced cardiac dysfunction while in the bathroom. Based on this number of cardiac arrest cases, it is estimated that approximately 17,000 people die annually while taking a bath. From these surveys, which evaluated bathing deaths, it has been clarified that bathing deaths are more common among the elderly people because three-fourths of the victims were aged 65 years and older [16, 19]. Looking at the onset by month, it is clear that the cases are many in winter when the temperature is very low [25], and few in the summer, moreover, the number of bathing-related deaths in January, was 10.7 times higher than that in August, which has the lowest reports of deaths [22]. Suzuki et al. [15] also conducted a broad surveillance program in three areas and estimated that the annual number of deaths during bathing is 19,000.

**Fig. 1:**
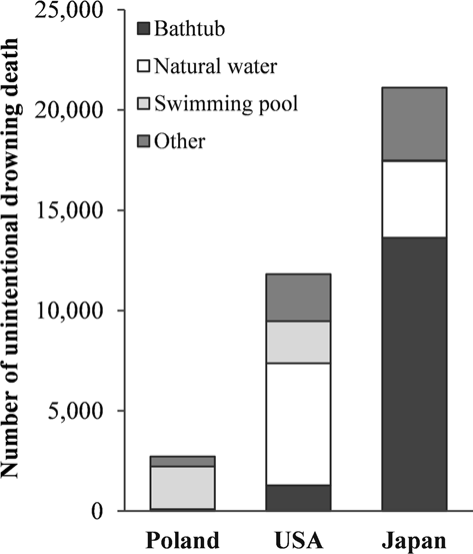
Distribution of accident locations in Poland, USA and Japan

Do bathing-related deaths frequently occur among the elderly population worldwide? Lin et al. [26] compared the unintentional drowning mortality rate for 3 years in 60 countries by age and accident location. Japan reported a relatively low mortality rate (0.9 deaths per 100,000 population) among younger adults aged 15–24 years, but had the highest mortality rate (19.0 deaths per 100,000 population) among the elderly aged 65+ years. Figure 1 shows the distribution of accident locations among the three countries with the highest number of victims from drowning deaths among the 60 countries [26]. Japan had the highest number of victims from drowning and highest incidence of bathtub accidents (64.6%). On the other hand, in the USA and Poland, drowning mostly occurred while swimming in pools, seas and rivers. The high number of deaths in bathtubs in winter among the elderly is a characteristic event in Japan [21]. These deaths, associated with bathing, are common among the elderly in winter and are a prominent accident in Japan.

### Surveys on JSB

To prevent Japanese bathing accidents, many surveys on bathing patterns (frequency, immersion depth, duration, time zone, etc.) and bathing environment (room temperature and hot water temperature) have been conducted. Kanda et al. [27] measured the temperature of the living rooms and dressing rooms of the detached houses of 42 elderly people living in North-Tokyo in winter, the average temperatures of these areas were only 15.0 °C and 13.5 °C , respectively, and the average hot water temperature was 40.8 °C. In the summer, the average temperature in the living room was 28.0 °C, that in the dressing room is 29.5 °C, and that of the average hot water temperature was 40.1 °C, emphasizing the need to improve the thermal environment of the bathroom and dressing room in winter. Upon measuring SBP fluctuations associated with bathing, it was found that the SBP rose sharply when the individuals were naked while staying in a cold dressing room in winter, and the average SBP was greatly affected by room temperature.

**Fig. 2:**
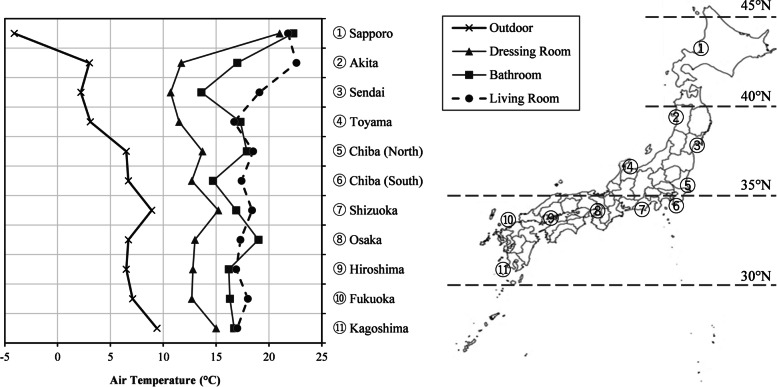
Average temperature by room and outdoor temperature in 11 regions in Japan during winter bathing. Adapted from Ohnaka et al. [10]

We measured the thermal environments (living room, bathroom, dressing room, and outdoor air) of 331 detached houses in 11 regions (Sapporo, Akita, Sendai, North Chiba, South Chiba, Shizuoka, Toyama, Osaka, Hiroshima, Fukuoka, and Kagoshima) every minute for 1 week in winter [10]. In addition, a questionnaire survey regarding bathing habits was conducted among the elderly people who were living in the area [28]. Figure 2 shows the temperature by room (average value for 10 min of bathing) during winter bathing by region [10]. In each area, the temperature in the living room was high, and while that in the dressing room was the lowest. In Sapporo, the average temperature in the dressing room and bathroom, when naked and in cold stress, were 21.0 °C and 22.3 °C, respectively, in other regions, the average temperature in the dressing room and bathroom were kept at 10–15 °C and 15–20 °C, respectively. Sapporo is geographically located more northward than other regions, and the outdoor temperature is very low in winter. Therefore, installation of heating systems widely practiced, and the temperatures in bathrooms and dressing rooms are relatively high. The relationship of drowning mortality rate with temperature of each room in the home was investigated by using a multivariate analysis. The only room temperature that showed a significant relationship with the drowning mortality rate by prefecture was the dressing room temperature. In Akita and Toyama, where the drowning mortality rate was high, the dressing room temperature was found to be low. It was shown that heating the dressing room was essential for preventing sudden death during bathing.

Takasaki et al. [29] conducted a questionnaire survey on bathing habits of 583 elderly people in four regions with different drowning mortality rates (low rate, Sapporo; medium rate, Osaka; high rate, Akita and Fukuoka). Sapporo and the other three regions were compared by conducting a logistics regression analysis; results showed that the winter bathing habits in Sapporo, the region with the lowest rate of mortality due to bathing accidents, are “less bathing,” “short stay in the bathroom,” “short soaking time in the bathtub” and “do not feel so cold while taking a bath.” They reported that if the elderly people develop the abovementioned bathing habits, it can effectively prevent fatal bathing accidents.

Yatsuzuka et al. [30] conducted an online questionnaire survey (4161 people) to determine the bathroom specifications, heating equipment, and winter bathing habits of individuals living in detached houses and apartments in warm and cold regions. Approximately 68.4% of respondents took a bath daily, indicating that the older the person, the more often they take a bath in the bathtub. In addition, those who complained that the bathroom was cold in winter had a higher water temperature, had less satisfaction with bathing, and had a higher incidence of physical discomforts (dizziness, stagger, and hot flashes) while in the bathroom.

In summary, elderly people in Japan have a habit of bathing in a cold dressing room/bathroom in winter almost every day from evening to night in hot water of 40 °C or higher by soaking in the water up to their shoulders, this finding shows that such bathing habits will increase the elderly people’s risk of illness.

### Physiological and subjective responses of the elderly related to JSB

The reason why bathing deaths are more common among the elderly in winter is thought to be due to the subjective and physiological characteristics of the elderly in relation to the thermal environment during bathing.

#### Effects of cold exposure on thermal sensation and BP in the dressing room

The dressing room is usually located outside the bathroom. The Japanese people first remove their clothes in the dressing room, and then enter the bathroom. In most Japanese detached houses, the dressing rooms and bathrooms are located on the north side and have poorly insulated windows [11]. Therefore, elderly people in Japan get naked in a cold dressing room before taking a bath in winter. Experiments were conducted to measure the thermal responses of the elderly and young people by exposing them to the cold of around 10 °C naked, and it was reported about 70 years ago that the elderly had less complaints of discomfort due to the cold [31]. However, no specific results were given. Therefore, Tochihara et al. [32] conducted an experiment comparing the feeling of coldness and warmth when elderly women and young women were exposed to cold (10 °C) or hot (35 °C) for 50 min while dressed in nightwear. Results showed that although there was no difference in the feeling of coldness and warmth between the two groups after 30 min or more, there were significantly fewer complaints of coldness in the elderly immediately after exposure to the cold room. This finding indicates that elderly people have delayed sensitivity to cold and are more likely to tolerate cold.

The abovementioned experiments represented passive methods in determining the comfortable temperature. In contrast, active determination of the comfortable temperature can be carried out using a certain method. Each person can be allowed to control the temperature of the artificial climate room, and the change in room temperature can be recorded. Collins et al. [33] used this method to determine the comfortable temperature for young people and older people over 70 years old, no significant difference in preferred temperature was observed between young and old people. However, elderly people had less ability to control the temperature, and the fluctuations of selected temperature for elderly people were significantly larger than that for young people. Thus, elderly people have decreased sensitivity to temperature changes. Ohnaka et al. [34] and Taylor et al. [35] also conducted experiments in an artificial climate room to select the preferred temperature for the elderly and young people. Although there was no age difference in the preferred temperature, elderly people were less likely to complain of discomfort due to cold or hot temperature. Compared with young people, elderly people cannot control air conditioning properly, a greater thermal stimulus (cold/heat) is required. This finding indicates that special consideration is required when selecting the high and low temperature ranges for elderly people, especially in cold environment, beyond the comfortable temperature range.

**Fig. 3:**
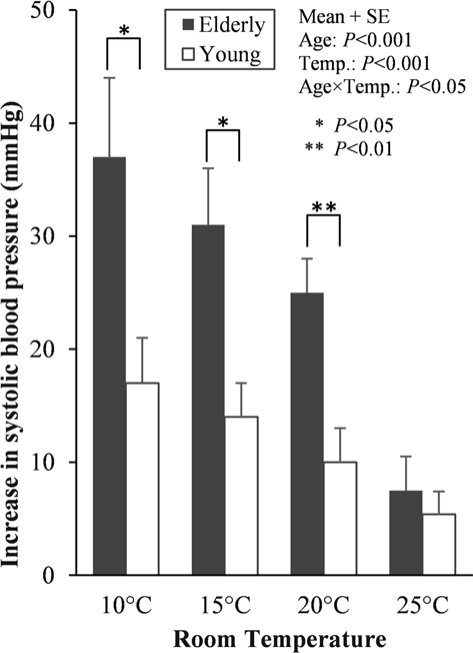
Age-related differences in systolic blood pressure increases in the dressing room under four room temperature conditions. Adapted from Tochihara et al. [39]

It is widely known that when the human body is exposed to cold, BP rises due to the vasoconstriction of the skin and internal organs, and this tendency is particularly strong in the elderly due to increase in vascular stiffening and deterioration of baroreceptor sensitivity [32, 36–38]. Tochihara et al. [39] focused on the age-related difference in SBP influenced by the dressing room temperature. The physiological response was measured while the elderly people were soaking in hot water (40 °C) for 8 min up to the shoulder under the condition of room temperature of 10 °C, 15 °C, 20 °C, and 25 °C. We showed the degree of increase in SBP due to cold exposure in the dressing room by group under each condition of room temperature as shown in Fig. 3. At room temperatures of 10 °C, 15 °C, and 20 °C, the degree of increase in BP was significantly greater in the elderly group; however, at room temperature of 25 °C, there was no difference between the groups. At a room temperature of 20 °C, the younger group demonstrated an average BP increase of 10 mmHg or less, while the elderly group demonstrated an average BP increase of 25 mmHg. Based on these findings, a room temperature of 20 °C was not sufficient for the elderly.

Recently, Tochihara et al. [38] investigated the age difference in physiological and subjective responses to a wide range of gradual ambient temperatures changes. We found no age difference in thermal sensation even though the BP of the elderly increased significantly during cold and rewarming periods. This gap could be explained by the blunted cutaneous thermal sensitivity thresholds in the elderly to warm and cold stimuli on their skin [40, 41].

In summary, elderly people are less likely to feel the coldness of the dressing room, and do not consider it as unpleasant. On the contrary, elderly people tend to exhibit a higher BP upon exposure to the cold, and they require higher room temperatures to prevent a rapid increase in BP. A large increase in physiological burden without awareness may lead to accidents among elderly people while taking a bath.

#### Effects of hot water immersion on subjective and physiological responses in bathtubs

Although few studies have reported the age difference in sensitivity with respect to the temperature of hot water, Tochihara et al. [39] compared the thermal sensation of eight elderly people and eight young people when they were soaked in hot water, with a temperature of 40 °C up to their shoulders for 8 min. On average, elderly people reported that they felt “slightly hot” while taking a bath, and young people reported that they felt “hot”, showing a significant age difference. Ono et al. [42] compared the warmth and discomfort of 11 elderly people and 10 young people when they were soaked in hot water at 39 °C or 42 °C up to the axilla for 8 min. The younger people showed a clear difference in thermal sensations between the hot water temperatures of 39 °C and 42 °C at the end of bathing, while the elderly did not report a difference in thermal sensations between the hot water temperatures. In addition, only young people complained of discomfort due to heat when the water temperature was 42 °C. Miwa et al. [43] also compared the degree of warmth and discomfort of 10 elderly and 10 young people when they were soaked in hot water at 41 °C up to the chest for 15 min. For young people, while taking a bath, the changed from “slightly hot” or “hot”, the elderly reported that it was almost “neutral.” The discomfort changed from “slightly comfortable” to “uncomfortable” in young people, and the declaration of elderly people was “comfortable” from beginning to end. This difference in sensitivity to hot water may lead to the tendency of elderly people to increase the temperature of hot water when bathing in winter and increase the water level in the bathtub [30].

Experiments have been reported in which the physiological responses associated with bathing in the elderly were measured and compared with the young (8 to 12 participants in each group). Nagasawa et al. [44] measured the BP and electrocardiogram during bathing at a hot water temperature of 40 °C for 10 min and for 15 min before and after the bathing, and they performed heart rate variability analysis. In younger people, parasympathetic activity decreased, BP gradually decreased, and heart rate increased during bathing. On the other hand, in elderly people, no change was observed in parasympathetic nerve activity during bathing, and it was reported that BP and heart rate increased immediately after bathing and began to decrease in about 4 min. Miwa et al. [45] compared the circulatory dynamics and thermoregulatory function when bathing in the axilla at a hot water temperature of 40 °C for 20 min. Elderly people had a sharp rise in SBP immediately after bathing and a drop during bathing. They also admit that the range of BP fluctuations is larger than that of young people, the increase in heart rate during bathing relatively small, and the increase in core temperature and sweating during bathing was also small.

Ono et al. [42] compared the age differences in physiological responses when bathing at a hot water temperature of 39 °C or 42 °C for 8 min. Focusing on the experiment with a hot water temperature of 42 °C, it was shown that the SBP and heart rate of the elderly increased immediately after bathing and decreased later. On the contrary, the heart rate of young people continued to increase while taking a bath. In addition, the amount of weight loss in the elderly was significantly small. Recently, Miwa et al. [43] compared the thermoregulatory response associated with bathing at a hot water temperature of 41 °C for 15 min between the elderly and young. It was revealed that the increase in tympanic temperature, local sweating (arm), and skin blood flow (forearm) while taking a bath was relatively smaller in the elderly.

The above experiments were carried out at moderate room temperature (20 °C to 26 °C), but Tochihara et al. [39] focused on the age difference due to the influence of bathroom/dressing room temperature. The physiological response when bathing in hot water at 40 °C for 8 min under room temperatures of 10 °C, 15 °C, 20 °C, and 25 °C was measured as shown in the previous section. The lower the room temperature in the dressing room/bathroom, the greater the degree of decrease in BP during bathing, which was particularly observed in the elderly. The elderly people were shown to have a greater decrease in BP when bathing in a cold room compared with younger people.

**Fig. 4:**
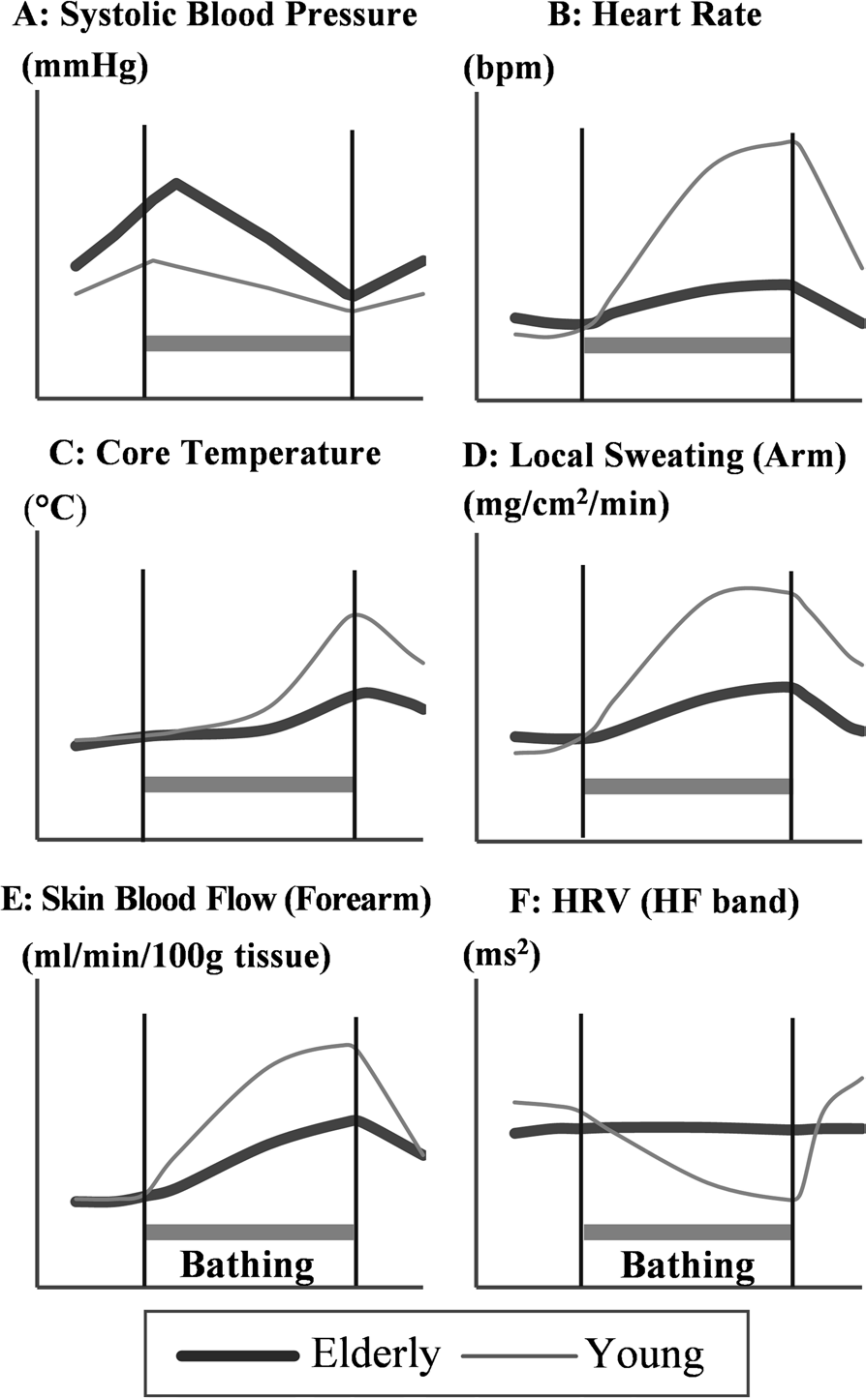
Age-related differences in physiological reactions during JSB (conceptual diagram). Adapted from Tochihara and Hashiguchi [46]

Fluctuations in the physiological response vary considerably depending on the hot water temperature, bathing time, room temperature, etc., the age difference in physiological fluctuations while performing JSB was summarized based on the following three experimental studies. The water temperature and immersion time reported by Nagasawa et al. [44], Miwa et al. [45], and Miwa et al. [43] were 40 °C for 10 min, 40 °C for 20 min and 41 °C for 15 min, respectively. The water level of the bath was almost shoulder level in all three experiments. A conceptual diagram of age-related differences in physiological reactions during JSB is shown in Fig. 4 [46].

The SBP (A) of the elderly increased once due to the sympathomimetic effect called “surprise reflex” immediately after bathing [20] and then decreased more rapidly compared with that of the young. On the contrary, the increase in heart rate (B), which is considered to be a compensatory function of the baroreceptor reflex to decrease the BP, of the elderly was much smaller than that of the young during bathing. It seems that the suppression of visceral parasympathetic nerve activity (F: heart rate variability; HF power) is less likely to occur in the elderly compared to the young [47]. That is, in the elderly, the BP and heart rate during bathing are not properly regulated by the autonomic nerves, resulting in a large decrease in BP of the elderly. It has been pointed out that this leads to “syncope” and the accompanying “drowning” [46]. In fact, it has been reported that more than 80% of people had their faces submerged in bathtubs at the scene of bathing deaths attended by paramedics [15].

Elderly people have a low increase in core temperature (C) and a small increase in sweating (D) and skin blood flow (E). The reason why the core temperature of the elderly is less likely to rise is that they generally have a larger amount of subcutaneous fat than the young and their body is less likely to warm up [45]. Furthermore, physiologically, the low sweating in the elderly was due to the delayed sweating start time and age-related decrease in sweat output per single sweat gland [48]. The small increase in cutaneous blood flow is due to the decrease in skin vasodilatory ability [48, 49].

In any case, elderly people prefer a long-term high-temperature bath and tend to immerse the shoulder because the physiological thermal effect associated with bathing is low and it is difficult to feel the heat of the hot water. This also seems to lead to an increase in bathing deaths among the elderly in winter.

### Prevention of sudden death during bathing

“The methods for preventing elderly bathing death in winter” were published by several institutions (e.g., Consumer Affairs Agency, Japan [50]), and were as follows: (1) warming the dressing room/bathroom, (2) avoiding heating the water (41 °C or less), (3) avoiding taking long baths (within 10 min), (4) taking a half-body bath rather than a full-body bath, (5) avoiding rapidly getting up from the bathtub to prevent a rapid decrease in hydrostatic pressure, (6) drinking water before and after bathing to prevent dehydration, (7) encouraging elderly people to call out diligently when taking a bath, and (8) avoiding taking a bath when not feeling well or after drinking alcohol.

Regarding the points numbered (2), (3), and (4), high water temperature bath and long bath are preferred when the room temperature is low [29, 30], and a half-body bath is not preferred because it is uncomfortable and unrealistic in the cold environments [51]. That is, many surveys and experiments revealed that items (2), (3), and (4) can be resolved by warming the dressing room/bathroom. Therefore, I examined the recommended and permissible values of the dressing room/bathroom temperature in winter, which is the key measures for preventing bathing death in the elderly in winter. The World Health Organization Guidelines for Housing and Health, published in 2018 [52], recommend a minimum room temperature of 18 °C in winter.

When examining the relationship between the health of the elderly and the thermal environment, the BP is often used as the evaluation of physiological strain as described above. Collins et al. [36] investigated the BP fluctuations in the elderly and young when exposed to a temperature of 6 °C to 15 °C for 2 h in winter while dressed in nightwear. At room temperature of 6 °C, the BP of young people tended to stabilize after increasing, while the BP of elderly people continued to increase. At a room temperature of 12 °C, the BP of young people hardly increased, while in the elderly, the BP increased significantly. From the viewpoint of BP fluctuations, it was reported that the room temperature of the elderly should be kept at 15 °C. Umishio et al. [53] conducted a large-scale winter housing survey from 2014 to 2017 to measure the BP and room temperature in the early morning and before bedtime of 2900 people (mean age 57 years). The room temperature at which SBP does not exceed 135 mmHg was examined by age group and gender by conducting a detailed analysis; results showed that a room temperature of 19 °C or higher is required for men in their 70s.

However, these standard values represent the room temperatures when loungewear is worn. On the contrary, what should be the permission temperature of the dressing room/bathroom when an individual is naked? Tochihara [5] conducted a bathing experiment in which 12 young men were immersed in a hot water temperature at 40 °C for 8 min under seven conditions of room temperature of 5 °C, 10 °C, 15 °C, 20 °C, 25 °C, 30 °C, and 35 °C. The BP increased sharply at room temperature of 5 °C, 10 °C, and 15 °C, and decreased at a room temperature of 35 °C. Therefore, the permissible room temperature range based on the BP was 20 °C to 30 °C. Furthermore, it has been reported that a room temperature of 20 °C or higher to prevent the increase in BP associated with bathing, and that the elderly should be kept warmer as shown in Fig. 3.

In summary, when bathing safely and comfortably in winter, the dressing room/bathroom temperature must be maintained at 20 °C or higher; for elderly people, it is safer to increase the room temperature by several degrees.

## Merits of JSB

There are, however, many research reports on the beneficial effects of JSB on sleep [54–61], self-rated health [58, 59, 62], rehabilitation [63, 64], fatigue [60, 62, 65], depression [66, 67], and BP [68].

Tokyo Gas Inc. [7] conducted a survey among 2600 people living in the Tokyo metropolitan area to determined their daily bathing habits in winter. The average current Japanese bathing habits were as follows: set temperature of hot water; 40–42 °C (71.8%), duration in bathroom; 26.4 min, immersion time in bathtub; 13.7 min, and bathing time; 20:00–23:00. Ishizawa et al. [62] reported that soaking in hot water bathing up to the shoulders was commonly practiced (83.3%) in Japan. Tai et al. [67] also conducted a survey among 1035 elderly participants living in Nara Prefecture to assess their bathing habits in winter. The mean bathing start time, bedtime, bathing duration and hot water temperature were 20:31, 22:31, 13 min, and 40.7 °C, respectively.

It is difficult to achieve a deep sleep, and the time taken to fall asleep is extended in old age [69]. In early studies of the effects of bathing on sleep the warm bath of 41–43 °C temperature in an immersion tank or bathtub, with immersion of the whole body up to mid-thorax or neck high for 30–90 min with a 10–30 min interim cooling down period were performed in 1980s [70–73]. They reported that warm baths could enhance slow-wave sleep, reduce rapid eye movement (REM) sleep, and decrease sleep onset latency (SOL) in heathy young adults. As a physiological mechanism by which passive body heating (PBH) brings a good sleep, it has been suggested that the mechanism by means of which PBH affects sleep is that warming promotes a subsequent sleep fall in core body temperature, mimicking the decrease in core body temperature seen in the hours proceeding habitual bedtime [74, 75]. Since 2000, many studies [55, 76] reported that foot baths as well as bathtub baths improve sleep quality. A footbath is especially recommended for people with disability who are unable to enjoy regular baths easily and safely. Kräuchi and colleagues [77, 78] revealed increased distal vasodilation, as indicated by the distal-proximal skin temperature gradient (DPG), and shorter SOL by PBH. They showed that a greater DPG could predict shorter SOLs in healthy individuals. Tai et al. [61] also showed similar results in an epidemiological study among 1094 elderly people (mean age, 72.0 years). They revealed that hot water bathing before bedtime in home settings is significantly associated with shorter actigraphic/self-reported SOL and higher DPG in a large-scale older population. They suggested that bathing for 1–3 h before bedtime showed significantly shorter SOL, and higher DPG 30 min after bedtime.

In the experiments performed in 1980s; however, the participants immersed themselves in the baths for more than 30 min, and their body core temperature increased about 1.6–2.6 °C, these values are higher than those practicing the JSB (about 0.6-1.0 °C). Therefore, their experiments did not directly show the effect of JSB on sleep. Thus, this article reviews the studies that assessed the effects of JSB on sleep as shown in Table 2. The only studies listed in the table are JSBs as defined below: water temperature, 40–41 °C; bathing duration, 10–30 min; immersion level, mid-thorax to neck; and bathing time, evening to night. Studies on the effects of showers and foot baths, bathing too hot, bathing too long, and bathing in the morning and early evening on sleep were excluded. However, studies conducted outside Japan, demonstrated that the bathing style is similar to the characteristics of JSB, are listed in the Table 2 [79–81]. Liao [84] reviewed the effects of bathing on body temperature and sleep regulation in the elderly from three studies including our study [54]. The review showed that bathing immersed to mid-thorax with 40–41 °C water for 10–30 min in the evening can increase the duration of slow-wave sleep in healthy elderly women, even in those with insomnia. Recently, Haghayegh et al. [85] reviewed by a meta-analysis to evaluate the effects of body heating by taking warm showers, foot baths or bathing on the quality of sleep in 13 studies, including our study [55]. They concluded that body heating in 40–42.5 °C water for a duration as short as 10 min scheduled 1–2 h before bedtime is associated with shortened SOL and increased sleep efficiency. The bathing conditions they considered to provide good sleep were in good agreement with the JSB above as we defined.

**Table 2:** Studies on the effects of JSB on sleep

Author (year)	Subjects		Bathing			Time Before bedtime	Sleep assessment	⧍Tc (°C)	Ta (°C)	Research objective
	*N*	Age (SD)	Tw (°C)	Duration (min)	Body site					
Horne and Shackll (1987) [71]	3M, 3F	21–33	41.0	30	Mid-thorax	**5.3 h**	PSG, Tc, SS	2	−	Timing
			41.0	30		2.3 h		2		
Dowdell and Javaheri (1992) [79]	7M	59.0 (4.5)	41.0	30	Upper- chest	2.5 h	PSG	2	19.5	Apnea/hypopnea
Dorsey et al. (1996) [80]	9F	65.1 (3.3)	40.3	30	Mid-thorax	1.5 h	PSG, Act, Tc, SS	0.9	−	Water temperature
	9F		**38.0**	30		1.5 h		0.2		
Dorsey et al. (1999) [81]	14F	60–73	40.3	30	Mid-thorax	1.75–2 h	PSG, Tc	0.7	−	Insomnia
Kanda et al. (1999) [54]	10M, 20F	20.5 (0.2)	40.7	10-	Neck	0.5h–	BM, Tc, SS	0.7	8–12	Field study
	13M, 17F	73.2 (0.9)	40.2	10-	Neck	0.5 h–		0.6		
Sung and Tochihara (2000) [55]	9F	21–40	40.0	20	Shoulder	0.5 h	PSG, BM, Tc, SS	1	10	Footbath
	9F	21–40	42.0	30	**Knee**	0.5 h		0	10	
Sung et al. (2000) [82]	8M	22.0 (3.0)	40.0	20	Shoulder	**0.3 h**	PSG, BM, Tc, SS	0.9	10	Timing
	8M		40.0	20	Shoulder	1.5 h		0.8	10	
Mishima et al. (2005) [56]	2M, 1F	76.9	40.0	30	Mid-thorax	2.0 h	DLMO, Act Tc, HRV	0.8	24	Dementia
Inagaki et al. (2007) [83]	6F	21.6 (0.6)	40.0	10	Neck	0.5 h	PSG, HRV Tc, SS	0.5	27	Timing
	6F		40.0	10	Neck	1.0 h		0.4	27	
	6F		40	10	Neck	2.0 h		0.4	27	

*N* number, *M* male, *F* female, *SD* standard deviation, *Tw* water temperature, *PSG* polysomnography, *Act* actigraphy, *Tc* core body temperature, *BM* body movement, *SS* subjective sleep sensation, *HRV* heart rate variability, *DLMO* dim-light melatonin-onset time, *Ta* air temperature

All studies except that of Inagaki et al. [83] involved experiments without the bathing (baseline nights) assigned as the control

Bold data is not JSB

Of the nine studies shown in Table 2, the one by Dowdell and Javaheri [79] did not report an improvement in sleep quality from baseline. The main cause was that the participants were patients with apnea/hypopnea syndrome. Other experiments that were conducted for comparison did not also report improvements in sleep quality due to the following reasons: the water temperature was low (38.0 °C [80];), the time before bedtime was too long (5.3 h [71];), PBH was footbath [55] and the time before bedtime was very short (0.3 h [82];). Bunnell et al. [72] investigated the effects of timing of bathing on sleep, namely morning (within 1 h of awakening), afternoon (10 h before bedtime), early evening (6 h before bedtime), and late evening (just before bedtime). They showed that sleep onset time was reduced by bathing, particularly during early evening bathing. However, the bathing duration (60 min immersion at 41 °C) was exceeded that of JSB. Sung et al. [82] compared the quality of post-bath sleep 1.5 h before bedtime with that just before bedtime, and showed that bathing just before bedtime did not improve sleep quality. Browman and Tepas [86] investigated the effects of different pre-sleep activities on all-night sleep. They reported that the SOL was clearly prolonged when performing exercise immediately before going to bed. Continued physical excitement prevents the onset of sleep. JSB performed just before bedtime may have caused a great burden on the sympathetic nervous system and suppressed the onset of REM sleep [71].

**Fig. 5:**
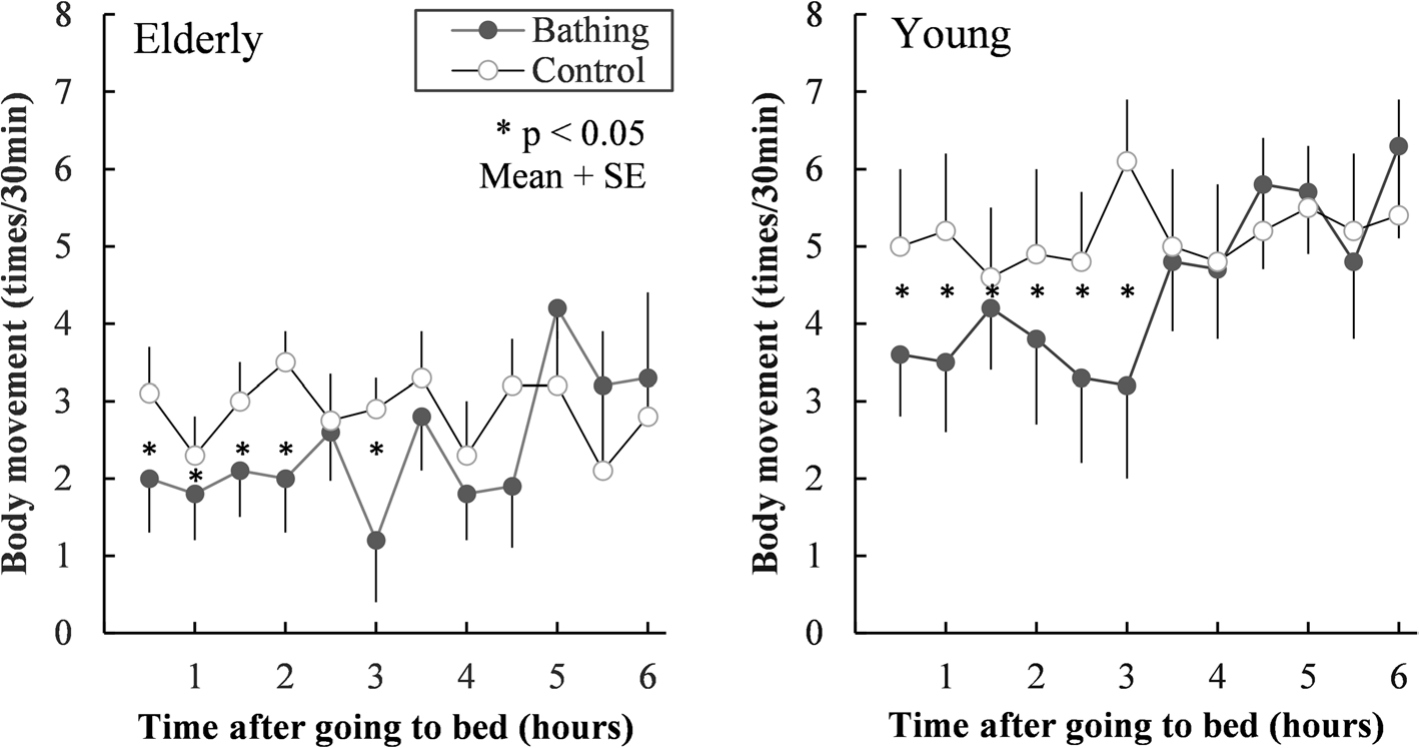
Number of body movements every 30 min during sleep in the elderly and the young. Comparison between the after bathing and the control. Adapted from Kanda et al. [54]

One of the effects that the elderly people expect from bathing is the promotion of sleep onset in winter [7]. However, although there have been studies conducted in the laboratory on the effects of bathing on sleep, none have investigated the effects of JSB at home on sleep objectively. We evaluated the quality of sleep after bathing in the elderly by measuring “body movement” [54]. This is because polysomnography (PSG) interferes with sleep, especially in the elderly. In winter, 30 elderly people aged 65–83 years (13 men and 17 women) and 30 young people aged 17–22 years (10 men and 20 women) were surveyed in their homes. The survey was conducted by comparing the days when people slept after bathing for at least 10 min, and the days when they did not take a bath. The measurement items were body movement number, rectal temperature, feeling of warmth and coldness, and feelings of sleepiness. The bedroom temperature was 8–12 °C for both age groups, and these temperatures were approximately the same as the average bedroom temperature (12.8 °C) in recently detached homes [11]. Figure 5 shows the number of body movements every 30 min during sleep for the elderly and young. Although the number of body movements in the young group tended to be higher than that in the elderly group as a whole, the number of body movements up to 3 h after bedtime in both groups was significantly higher after bathing than in the control group. It was found that bathing in winter improved the quality of sleep in the first half of sleep. According to the reports received after waking up, elderly people often reported that “sleep onset”, and “deep sleep” improved after bathing.

JSB can also ameliorate sleep disturbances in patients with insomnia [80, 81] and dementia [56]. JSB habits appear to have led to improvements in self-rated health [58, 59] and depressive symptoms [62, 66, 67, 87]. In an epidemiological study, Aritake-Okada et al. [57] investigated the relationship between daytime sleepiness and coping with regular behavior (having a bath, reading or listening to music, eating and drinking, etc.) to obtain adequate sleep in 24,686 general adults in Japan. They reported that “having a bath” was the most prevalent non-pharmacological self-management practice, especially for the elderly. Bathing reduced the daytime sleepiness complaints, and was associated with improved quality of nighttime sleep. On the contrary, exercising (USA) and reading (Canada) were most actively adopted as self-management practice for sleeping by participants in studies conducted outside Japan [57]. This may be attributable to the unique characteristics of JSB. Since sleep disturbance is known to be associated with the onset of mental disorders such as depression [88, 89], JSB can be a favorable practice that improves mental symptoms such as depression. Yagi et al. [66] investigated the association between a higher frequency of hot water bathing and lower depressive symptoms. They found that participants (4466 Japanese elderly) who took baths more frequently were less likely to be depressed after 3 years. In particular, it was shown that bathing in winter has a great effect of suppressing the onset of depression. Yagi et al. [87] also indicated that a high frequency of tub bathing is associated with lower onset of functional disability including depression among Japanese older adults. Recently, Tai et al. [67] showed interesting results from a logistic regression analysis of JSB habits and depression symptoms. They divided 1103 older volunteers by days of taking a bath in 2-day survey into three groups. Compared with the both-day bathing group, the either-day and no bathing groups were significantly associated with higher odds of depression symptoms. Moreover, they showed that shorter time before bedtime was associated with lower odds of depression symptoms.

In recent decades, the proportion of JSB has decreased, and the number of bathing styles that only require showering has increased (4.5%), especially in younger ages (men in their 20s; 14.5%), and the bathing style has become more westernized [7]. However, Yasuda et al. [60] reported the superior efficacy of JSB compared with showering. The study included were 18 university students who only showered on a daily basis, half of them continued to take a shower, while the other half adopted the JSB (10 min immersion at 41 °C water) for 2 weeks. They concluded that changing the bathing style from shower to JSB improves the participant’s quality of sleep and work efficiency. Moreover, the percentage of Japanese who like JSB remained high at 80%, and the dressing room temperature in winter in Japan (13.0 °C average value from 2190 detached houses) remained low [11] compared with those in Europe and the USA [90]. Future research related to JSB on human health and comfort is still desired.

In summary, JSB, which involves immersion of the body from mid-thorax to neck in 40–41 °C water for 10–30 min at 0.5–2.0 h before bedtime, can improve sleep quality, especially shortening of SOL. JSB is a simple and low-cost non-pharmacological measure of sleep difficulties in winter, especially for the elderly. Moreover, JSB can be a favorable practice that could prevent the onset of mental disorders such as depression.

## Conclusions

Japanese people like hot water bathing and often soak their body up to the shoulders in deep bathtubs for a long time in the evening to night. Experimental and epidemiological studies have shown that JSB shortens SOL and improves sleep sensation in winter. JSB is a simple and low-cost non-pharmacological measure for improving sleeping difficulty in winter, especially for the elderly. Moreover, JSB can be a favorable practice that could prevent the onset of mental disorders such as depression. On the contrary, drowning, which commonly occurs in bathtubs, is the most common cause of accidental death at home in Japan. It is estimated that approximately 19,000 Japanese individuals die annually while taking a bath, most accidents occur in winter, and most victims are elderly. Elderly people are likely to prefer a higher-risk JSB because the room temperature inside the house during winter in Japan, especially the dressing room/ bathroom temperature, is very low. Since the physiological thermal effect of the elderly associated with bathing is relatively lower among the elderly than the young, the elderly prefer to take a long hot bath. This elderly’s favorite style of JSB results in larger increased BP in dressing rooms and larger decreased in BP during hot bathing. A sudden drop in BP while immersed in the bathtub leads to fainting and drowning. Furthermore, elderly people are less likely to feel cold or hot water, therefore, it is difficult to take appropriate measures. For safe and comfortable winter bathing, the dressing room temperature needs to be maintained at 20 °C or higher, and several degrees higher would be recommended for the elderly.

## Data Availability

All data generated or analyzed during this study are included in this published article.

## Abbreviations

BP: Blood pressure
DPG: Distal-proximal skin temperature gradient
HF: High frequency
JSB: Japanese-style bathing
PBH: Passive body heating
PSG: Polysomnography
REM: Rapid eye movement
SBP: Systolic blood pressure
SOL: Sleep onset latency
